# Liver disease and 30-day mortality after colorectal cancer surgery: a Danish population-based cohort study

**DOI:** 10.1186/1471-230X-13-66

**Published:** 2013-04-15

**Authors:** Jonathan Montomoli, Rune Erichsen, Christian Fynbo Christiansen, Sinna Pilgaard Ulrichsen, Lars Pedersen, Tove Nilsson, Henrik Toft Sørensen

**Affiliations:** 1 Department of Clinical Epidemiology, Aarhus University Hospital, Olof Palmes Allé 43-45, Aarhus N DK-8200, Denmark

**Keywords:** **L**iver disease, Colorectal neoplasms, Surgery, Mortality, Epidemiology

## Abstract

**Background:**

Colorectal cancer (CRC) is common, with surgery as the main curative treatment. The prevalence of chronic liver disease has increased, but knowledge is limited on postoperative mortality in patients with liver disease who undergo CRC surgery. Hence, we examined 30-day mortality after CRC surgery in patients with liver disease compared to those without liver disease.

**Methods:**

We used medical databases to conduct a nationwide cohort study of all patients undergoing CRC surgery in Denmark from 1996 through 2009. We further identified patients diagnosed with any liver disease before CRC surgery and categorized them into two cohorts: patients with non-cirrhotic liver disease and patients with liver cirrhosis. Patients without liver disease were defined as the comparison cohort. Using the Kaplan-Meier method, we computed 30-day mortality after CRC surgery in each cohort. We used a Cox regression model to compute hazard ratios as measures of the relative risk (RR) of death, controlling for potential confounders including comorbidities. In order to examine the impact of liver disease in different subgroups, we stratified patients by gender, age, cancer stage, cancer site, timing of admission, type of surgery, comorbidity level, and non-hepatic alcohol-related disease.

**Results:**

Overall, 39,840 patients underwent CRC surgery: 369 (0.9%) had non-cirrhotic liver disease and 158 (0.4%) had liver cirrhosis. Thirty-day mortality after CRC surgery was 8.7% in patients without liver disease and 13.3% in patients with non-cirrhotic liver disease (adjusted RR of 1.49 95% confidence interval (CI): 1.12-1.98). Among patients with liver cirrhosis, mortality was 24.1%, corresponding to an adjusted RR of 2.59 (95% CI: 1.86-3.61). The negative impact of liver disease on postoperative mortality was found in all subgroups.

**Conclusions:**

Pre-existing liver disease was associated with a markedly increased 30-day mortality following CRC surgery.

## Background

Prevalence of liver diseases is increasing worldwide, and fatty liver and liver cirrhosis are known risk factors for colorectal cancer (CRC) [1,2]. Among the available treatments for CRC, surgical excision of the primary tumor remains the only curative approach [3] and liver disease patients who have CRC surgery may be at increased risk of postoperative complications and death, related to effects of anesthesia, bleeding during surgery, infections, and subsequent multi-organ failure [4].

However, only a few studies have addressed the association between liver disease and mortality following colorectal surgery [5-10], and only one focused on mortality after CRC surgery [5]. Former studies reported in-hospital and 30-day mortalities in liver disease patients ranging from 6% to 41% after colorectal surgery, compared with 1% to 5% in patients without liver disease. The majority of former studies has been based on data from referral centers [5,8,9] and did not include patients with non-cirrhotic liver disease [5,7-10]. Moreover, they have been hampered by small study populations [5,8,9], lack of information on comorbidity and surgery [5,8], lack of comparison cohorts of patients without liver disease undergoing same type of surgery [5,8,9], and restriction to in-hospital mortality [7,9,10]. The comorbidity data in former studies were limited by incomplete information as only diagnoses recorded within a short period before surgery were available [6,7,9,10].

Accurate data on mortality in patients with liver disease undergoing CRC surgery are needed to better understand the prognostic effect of liver disease in CRC patients. Such results also may help to optimize perioperative care.

We therefore conducted a nationwide cohort study investigating 30-day mortality after CRC surgery in patients with liver disease compared to those without liver disease.

## Methods

This cohort study was conducted within the entire Danish population which accumulates to 6.8 million people in the study period from January 1, 1996 through December 31, 2009. The National Health Service provides tax-funded medical care, including CRC surgery, for all Danish residents. Since 1968, a unique civil registration number (CPR number) has been assigned to all Danish residents at birth or upon immigration [11]. The CPR number allows accurate record linkage at an individual level among all Danish registries. The study was approved by the Danish Data Protection Agency. According to Danish law, the study did not require approval from the health research ethics committee system.

### Study cohort

We included all patients with a diagnosis of CRC who underwent first-time CRC surgery during the study period. Patients with a CRC diagnosis (see Additional file 1 for diagnosis codes) were identified using the Danish Cancer Registry (DCR), which contains records of all incident cases of malignant neoplasms in Denmark since 1943 [12]. Data recorded for each individual include method of cancer verification, cancer stage, and place of residence on the date of cancer diagnosis. Tumors registered after January 1, 1978 have been reclassified according to the *International Classification of Diseases, 10*^*th*^*revision* (ICD-10).

We used the CPR number to link CRC patients identified in the DCR to the Danish National Registry of Patients (DNRP) to obtain information about comorbidities and surgery. The DNRP includes information on all non-psychiatric hospitalizations since 1977 and on outpatient contacts since 1995 [13]. Diagnoses have been recorded according to the ICD-8 until 1993 and according to the ICD-10 thereafter. Each record includes the dates of hospital admission and discharge, up to 20 discharge diagnoses, the type of admission (acute or elective), and information about surgery, including type and date of surgical procedure. Since 1996, surgical procedures have been coded according to the NOMESCO (Nordic Medico-Statistical Committee) Classification of Surgical Procedures [14]. We therefore chose 1996 as the beginning of our study period.

As the indication for surgery is not coded in the DNRP, we defined CRC surgery as a procedure involving colorectal surgery performed during a hospitalization where CRC was listed as a diagnosis in the DNRP (see Additional file 1 for codes). We categorized CRC surgery into groups according to type of the first recorded procedure. “Radical resection” included surgeries with the intention of eradicating the primary tumor, such as partial and total resections of the colon and/or rectum. This group was further divided into laparoscopic and open surgery. “Non-resectional procedures” included colostomy, stent placement, or excision of a very small part of the colon (see Additional file 1 for codes). For each patient we reported the timing of the admission as elective or acute using the information about type of the hospitalization in the DNRP.

We classified CRCs with local spread at the time of first diagnosis as “localized” and those with regional and/or distant metastases as “non-localized” (see Additional file 1 for codes).

### Liver disease

We used the DNRP to identify patients with a diagnosis of liver disease (see Additional file 1 for diagnosis codes) before the CRC surgery date. Liver disease patients were divided into two different cohorts: patients with non-cirrhotic liver disease and patients with liver cirrhosis [15]. Non-cirrhotic liver disease included all liver disease diagnoses except liver cirrhosis, *eg*, viral hepatitis, alcoholic hepatitis, non-alcoholic fatty liver disease, or primary biliary cirrhosis. Patients with no history of liver disease prior to CRC surgery were defined as the comparison cohort.

### Comorbidity

We used the DNRP to compute Charlson Comorbidity Index scores to quantify the burden of comorbidity [16]. The Charlson Comorbidity Index includes 19 diseases, each assigned a score between one and six. The sum of the individual scores represents a measure of a patient’s level of comorbidity. We identified the diseases in the Charlson Comorbidity Index using ICD-8 and ICD-10 diagnosis codes [17], excluding mild and severe liver disease, CRC, CRC metastases, secondary liver cancers, and hepatocellular carcinoma. We classified patients as having a low (score = 0), a moderate (score = 1-2), or a high comorbidity level (score ≥ 3). In addition, we obtained information on hospital diagnoses of non-hepatic alcohol-related disease, defined as alcohol abuse or alcohol-related diseases disregarding alcoholic liver disease [18], and presence of gastric or esophageal varices (see Additional file 1 for relevant codes).

### Mortality data

We followed all CRC patients from the date of CRC surgery until death, emigration, or 30 days, whichever came first. Date of death or emigration was obtained from the Civil Registration System, which tracks the vital status and residence of all Danish residents and is updated daily [19].

### Statistical analyses

The Kaplan-Meier method was used to compute 30-day mortality after CRC surgery in each patient cohort overall and to consider colon and rectal cancer separately. Moreover, we stratified 30-day mortality in each cohort by period of CRC surgery (1996-2002 or 2003-2009) and timing of admission (acute or elective). We used a Cox regression model to compute hazard ratios as a measure of the relative risk (RR) of death and 95% confidence intervals (CIs), comparing 30-day mortality after surgery among CRC patients in each liver disease cohort to that of the comparison cohort of CRC patients without liver disease. In the first analysis, we controlled for gender, age, timing of admission, type of surgery, cancer stage, comorbidity level, and non-hepatic alcohol-related disease. The proportional hazard assumption was checked graphically and found appropriate.

Next, to examine the impact of liver disease on 30-day mortality after CRC surgery in subgroups within each cohort, we stratified the analysis by gender, age category (0-59, 60-69, 70-79, and 80+ years), comorbidity level (low, moderate, and high), cancer site (colon, rectum, or both), stage (localized, non-localized or stage unknown), timing of admission (acute or elective), type of surgery (open radical resection, laparoscopic radical resection, or non-resectional procedure), and non-hepatic alcohol-related disease (yes or no).

## Results

### Descriptive data

We identified 39,840 CRC patients undergoing CRC surgery. Of these, 369 (0.9%) had non-cirrhotic liver disease and 158 (0.4%) had liver cirrhosis. Median age at CRC surgery was 72 years among patients without liver disease, 69 years among patients with non-cirrhotic liver disease, and 67 years among those with liver cirrhosis. Among non-cirrhotic liver disease patients, 60 (16.3%) had alcoholic hepatitis, 49 (13.3%) had viral hepatitis, 34 (9.2%) had non-alcoholic fatty liver disease, 4 (1.1%) had primary biliary cirrhosis, and 222 (60.2%) had other non-cirrhotic liver diseases.

Of patients with non-cirrhotic liver disease and liver cirrhosis 37% had acute admission, compared to 32% in the comparison cohort of patients without liver disease (Table 1). CRC patients with liver disease, especially those with liver cirrhosis, were more likely to have comorbid conditions, including non-hepatic alcohol-related disease, than patients without liver disease (Table 1). The higher comorbidity level in patients with liver disease persisted when stratified by cancer stage. For instance, among patients with non-localized CRC, a high level of comorbidity was found in 4.0% of the patients without liver disease, in 8.7% of those with non-cirrhotic liver disease, and in 12.0% of those with liver cirrhosis (see Additional file 2). Furthermore, type of surgery differed, with non-resectional procedures performed in less than 12% of patients with non-cirrhotic liver disease or without liver disease and in approximately 17% of cirrhotic patients (Table 1).

**Table 1:** Characteristics of patients with and without liver diseases undergoing colorectal cancer surgery in Denmark, 1996-2009

**Subgroups**	**No liver disease**	**Non-cirrhotic**	**Liver cirrhosis**
	**N (%)**	**liver disease**	**N (%)**
		**N (%)**	
**Gender:**			
- Male	20,097 (51.1%)	188 (50.1%)	105 (66.5%)
- Female	19,216 (48.9%)	181 (49.1%)	53 (33.5%)
**Age (years):**			
- 0-59	7,046 (17.9%)	75 (20.3%)	39 (24.7%)
- 60-69	10,083 (25.7%)	116 (31.5%)	55 (34.8%)
- 70-79	13,169 (33.5%)	113 (30.6%)	50 (31.6%)
- 80+	9,015 (22.9%)	65 (17.6%)	14 (8.9%)
**Cancer site:**			
- Colon	25,905 (65.9%)	264 (71.5%)	100 (63.3%)
- Both colon and rectum	72 (0.2%)	1 (0.3%)	0 (0.0%)
- Rectum	13,336 (33.9%)	104 (28.2%)	58 (36.7%)
**Timing of admission:**			
- Acute	12,633 (32.1%)	137 (37.1%)	59 (37.3%)
- Elective	26,602 (67.7%)	231 (62.6%)	99 (62.7%)
- Missing	78 (0.2%)	1 (0.3%)	0 (0%)
**Cancer stage:**			
- Localized	17,044 (43.4%)	163 (44.2%)	65 (41.1%)
- Non-localized	18,863 (48.0%)	182 (49.3%)	76 (48.1%)
- Stage unknown	3,406 (8.6%)	24 (6.5%)	17 (10.8%)
**Surgery:**			
- Laparoscopic radical resection	3,483 (8.9%)	35 (9.5%)	10 (6.3%)
- Open radical resection	31,278 (79.5%)	293 (79.4%)	122 (77.2%)
- Non-resectional procedures	4,552 (11.6%)	41 (11.1%)	26 (16.5%)
**Comorbidity level:**			
- Low	24,301 (61.8%)	167 (45.3%)	60 (38.0%)
- Moderate	11,573 (29.4%)	145 (39.3%)	65 (41.1%)
- High	3,439 (8.8%)	57 (15.4%)	33 (20.9%)
**Non-hepatic alcohol-related disease**^†^	582 (1.5%)	46 (12.5%)	54 (34.2%)
**Gastric and esophageal varices**	19 (0.1%)	2 (0.5%)	29 (18.4%)
**Distribution of the diseases included in the modified Charlson Comorbidity Index:**			
Myocardial infarction	2,282 (5.8%)	15 (4.1%)	10 (6.3%)
Congestive heart failure	1,918 (4.9%)	21 (5.7%)	7 (4.4%)
Peripheral vascular disease	1,622 (4.1)	27 (7.3%)	13 (8.2%)
Cerebrovascular disease	3,348 (8.5%)	46 (12.5%)	15 (9.5%)
Dementia	359 (0.9%)	7 (1.9%)	3 (1.9%)
Chronic pulmonary disease	2,864 (7.3%)	54 (14.6%)	19 (12.0%)
Connective tissue disease	1,064 (2.7%)	25 (6.8%)	5 (3.2%)
Ulcer disease	2,121 (5.4%)	34 (9.2%)	30 (19.0%)
Uncomplicated type 1 and 2 diabetes	2,173 (5.5%)	42 (11.4%)	29 (18.4%)
Hemiplegia	71 (0.2%)	2 (0.5%)	0
Moderate to severe renal disease	568 (1.4%)	11 (3.0%)	9 (5.7%)
Diabetes with end organ damage	922 (2.4%)	20 (5.4%)	16 (10.1%)
Any tumor^*^	3,594 (9.1%)	39 (10.6%)	15 (9.5%)
Leukemia	101 (0.3%)	1 (0.3%)	1 (0.6%)
Lymphoma	214 (0.5%)	2 (0.5%)	3 (1.9%)
Metastatic solid tumor^§^	433 (1.1%)	8 (2.2%)	2 (1.3%)
AIDS	7 (<0.1%)	1 (0.3%)	0 (0.0%)

^*^ Colorectal cancer and hepatocellular carcinoma were excluded.

^†^ Including both diagnoses of alcohol abuse and non-hepatic alcohol-related diseases.

^§^ Colorectal cancer metastases and secondary liver cancers were excluded.

### Postoperative mortality

**Figure 1:**
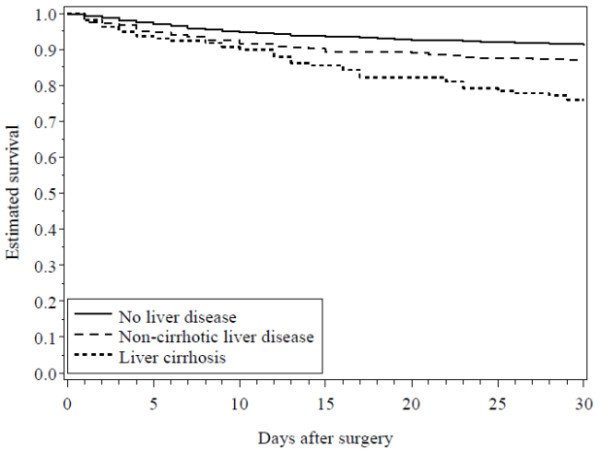
Crude 30-day survival curves for patients undergoing colorectal cancer surgery without liver disease, with non-cirrhotic liver disease, and with liver cirrhosis.

Thirty-day mortality was 13.3% in patients with non-cirrhotic liver disease and 24.1% among patients with liver cirrhosis, compared to 8.7% in patients without liver disease (Table 2). Moreover, survival among patients with non-cirrhotic liver disease seems to differ from that among patients with liver cirrhosis beyond the first week after CRC surgery (Figure 1). Compared with the cohort of CRC patients without liver disease, the adjusted RR was 1.49 (95% CI: 1.12-1.98) for non-cirrhotic liver disease and 2.59 (95% CI: 1.86-3.61) for patients with liver cirrhosis (Table 2). There was no substantial difference in the impact of liver disease on mortality in the 1996-2002 period and the 2003-2009 period (data not shown). Notably, the 30-day mortality among patients with acute admission was as high as 16.3% for those without liver disease, but increased to 24.1% among patients with non-cirrhotic liver disease and 35.6% among those with liver cirrhosis. Corresponding results for CRC patients electively admitted were 5.1% for patients without liver disease, 6.9% for those with non-cirrhotic liver disease, and 17.2% for CRC patients with liver cirrhosis (see Additional file 3).

**Table 2:** Relative risk (RR) and 30-day mortality after colorectal cancer surgery in patients with and without liver disease

**Cancer site**	**Patients**	**Deaths within 30 days**	30-day mortality %*	**RR (95% CI)**	
		**N**	**(95% CI)**	**Crude**	**Adjusted**
**Colorectal cancer**^**§**^					
- No liver disease	39,313	3,432	8.7 (8.4-9.0)	1.00	1.00
- Non-cirrhotic liver	369	49	13.3 (9.8-17.8)	1.56 (1.18-2.07)	1.49 (1.12-1.98)
disease					
- Liver cirrhosis	158	38	24.1 (16.7-33.9)	2.93 (2.13-4.03)	2.59 (1.86-3.61)
**Colon cancer**					
- No liver disease	25,905	2,569	9.9 (9.5-10.3)	1.00	1.00
- Non-cirrhotic liver	264	38	14.4 (10.2-20.1)	1.50 (1.09-2.06)	1.45 (1.05-2.00)
disease					
- Liver cirrhosis	100	27	27.0 (17.3-40.6)	2.90 (1.99-4.24)	2.50 (1.68-3.70)
**Rectal cancer**					
- No liver disease	13,336	857	6.4 (6.0-6.9)	1.00	1.00
- Non-cirrhotic liver	104	11	10.6 (5.6-19.3)	1.68 (0.93-3.04)	1.66 (0.91-3.02)
disease					
- Liver cirrhosis	58	11	19.0 (9.8-34.8)	3.14 (1.73-5.68)	2.84 (1.52-5.30)

RR, relative risk; CI, confidence interval.

* Calculated using the Kaplan-Meier method.

^§^ Overall colorectal cancers patients including patients with both colon and rectal cancers.

Thirty-day mortality was higher after colon cancer surgery than after rectal cancer surgery in all cohorts. Still, both colon and rectal cancer patients with liver disease had higher mortality than patients without liver disease (Table 2).

Table 3 shows adjusted RRs of 30-day mortality after CRC surgery for patients with liver disease stratified into subgroups. The impact of liver disease on 30-day mortality after surgery in CRC patients did not differ substantially between genders or within subgroups of patients with low and moderate comorbidity levels, different CRC stage, and elective or acute admission. Nevertheless, for patients with non-cirrhotic liver disease who underwent CRC surgery at an age of 60 years or younger, the RR was particularly high (adjusted RR = 2.71; 95% CI: 1.25-5.89). In patients with non-hepatic alcohol-related disease, the impact of liver disease on mortality was less pronounced for non-cirrhotic liver disease patients (adjusted RR = 1.30; 95% CI: 0.94-1.81) than for those with liver cirrhosis (adjusted RR = 3.37; 95% CI: 2.30-4.92). In addition, the impact of liver cirrhosis appeared more pronounced among patients less than 80 years old as in older patients whereas liver cirrhosis had little impact on patients undergoing non-resectional procedures (adjusted RR = 1.74; 95% CI: 0.82-3.67). Finally, the impact of liver disease was limited in patients with a high comorbidity level for both non-cirrhotic liver disease (adjusted RR = 1.17; 95% CI: 0.62-2.19) and liver cirrhosis (adjusted RR = 1.47; 95% CI: 0.68-3.15).

**Table 3:** Relative risk of 30-day mortality after colorectal cancer surgery in subgroups of patients with liver disease

**Subgroups**	**Patients N**	**No liver disease (reference cohort)**	Non-cirrhotic liver disease Adjusted* RR (95%CI)	Liver cirrhosis Adjusted* RR (95%CI)
**Gender:**				
- Male	20,390	1.00 (ref)	1.37 (0.88-2.13)	2.21 (1.20-4.01)
- Female	19,450	1.00 (ref)	1.56 (1.08-2.26)	2.82 (1.90-4.20)
**Age at colorectal surgery (years):**				
- 0-59	7,160	1.00 (ref)	2.71 (1.25-5.89)	3.53 (1.53-8.13)
- 60-69	10,254	1.00 (ref)	1.56 (0.83-2.95)	3.61 (2.00-6.52)
- 70-79	13,332	1.00 (ref)	1.10 (0.65-1.87)	2.37 (1.40-4.02)
- 80+	9,094	1.00 (ref)	1.61 (1.01-2.56)	1.14 (0.36-3.59)
**Timing of admission:**				
- Acute	12,829	1.00 (ref)	1.57 (1.11-2.22)	2.48 (1.59-3.88)
- Elective	26,932	1.00 (ref)	1.39 (0.85-2.28)	2.79 (1.70-4.57)
**Cancer stage:**				
- Localized	17,272	1.00 (ref)	1.16 (0.65-2.06)	3.49 (2.06-5.93)
- Non-localized	19,121	1.00 (ref)	1.71 (1.20-2.43)	2.42 (1.49-3.94)
- Stage unknown	3,447	1.00 (ref)	1.41 (0.58-3.42)	2.24 (0.91-5.50)
**Surgery:**				
- Laparoscopic radical resection	3,528	1.00 (ref)	NA	6.82 (1.48-31.45)
- Open radical resection	31,693	1.00 (ref)	1.41 (0.99-1.98)	3.01 (2.05-4.40)
- Non-resectional procedures	4,619	1.00 (ref)	1.91 (1.14-3.20)	1.74 (0.82-3.67)
**Comorbidity level:**				
- Low	24,528	1.00 (ref)	1.62 (0.97-2.70)	3.41 (1.97-5.91)
- Moderate	11,783	1.00 (ref)	1.60 (1.07-2.41)	3.14 (1.91-5.16)
- High	3,529	1.00 (ref)	1.17 (0.62-2.19)	1.47 (0.68-3.15)
**Non-hepatic alcohol-related disease**^**†**^**:**				
- Yes	682	1.00 (ref)	1.30 (0.94-1.81)	3.37 (2.30-4.92)
- No	39,158	1.00 (ref)	2.61 (1.43-4.76)	1.67 (0.88-3.10)

RR, Relative Risk; CI, Confidence Interval; NA, Not Applicable.

* Mutually adjusted for gender, age, timing of admission, cancer stage, surgery, comorbidity level, and non-hepatic alcohol-related disease.

^†^ Including both diagnoses of alcohol abuse and non-hepatic alcohol-related diseases.

## Discussion

We found that patients with non-cirrhotic liver disease or liver cirrhosis had a substantially higher postoperative 30-day mortality after colon and rectal cancer surgery than patients without liver disease. The association between non-cirrhotic liver disease and postoperative mortality appeared most pronounced among patients aged 60 years or younger. The impact of liver disease on mortality is evident among patients with low and moderate comorbidity levels, different CRC stage, and different timing of admission (acute vs. elective). However, among patients with a high level of comorbidity, we found a less pronounced impact of liver disease on mortality.

Thus, our data extend former research on postoperative mortality in patients with liver disease and CRC by using a population-based sample and, furthermore, particularly by evaluating the influence of other comorbidities and CRC stage and site. Only one cohort study based on data from a single US hospital in 2003 focused on mortality after CRC surgery in 72 patients with liver cirrhosis [5]. Of these, 49% had alcohol-related liver cirrhosis and the 30-day mortality after CRC surgery was 13%. Among patients with the most severe cirrhotic disease, identified as Child-Pugh class C, the postoperative mortality was 28% [5]. However, RRs could not be estimated because the study did not include a comparison cohort of patients without liver disease.

Other previous studies included patients undergoing colorectal surgery for non-CRC indications and did thus not estimate the impact of liver disease on postoperative mortality related to CRC alone.

Recently, Meunier *et al*. reported a 26% in-hospital mortality among 41 patients with liver cirrhosis undergoing colorectal surgery [9]. Of these patients, 39 had an alcoholic etiology and 35 received surgery for CRC. The results are supported by our finding showing that alcoholic liver cirrhosis represents an additional negative prognostic factor for patients undergoing CRC surgery [20,21].

Nguyen *et al.* reported a 29% in-hospital mortality after colorectal surgery among patients with liver cirrhosis complicated by portal hypertension and 14% in patients with compensated liver cirrhosis [10]. After stratification by acuity of presentation (elective vs. nonelective), in-hospital mortality was 1.8% vs. 9.1% among patients without liver cirrhosis, 7.2% vs. 20.9% among those with liver cirrhosis without portal hypertension, and 18.6% vs. 35.8% among those with liver cirrhosis with portal hypertension. Consequently, the impact of liver cirrhosis on mortality was higher among patients with elective admission (adjusted odds ratio = 3.91; 95% CI: 3.12-4.90) than in patients who had non-elective admission (adjusted odds ratio = 2.40; 95% CI: 2.07-2.79) [10]. Our results confirmed higher mortality among patients acutely admitted compared with those with an elective admission, especially among patients with liver cirrhosis. Yet, we did not show any major difference in adjusted RR between acute vs. elective admission. Finally, Ghaferi *et al*. analyzed 30-day mortality after colorectal surgery in about 1,500 patients with chronic liver disease, including both non-cirrhotic and cirrhotic diseases, and compared it to postoperative mortality in a group of 30,000 patients without liver disease. Patients with chronic liver disease had a postoperative mortality of 21.5% compared to an overall mortality of 3.2% in the control group [6]. Again, these results confirm that mortality among patients with liver disease – particularly those with complicated liver cirrhosis – is higher than in patients without liver disease. Unfortunately, none of the previous studies included non-cirrhotic liver disease as an individual group, and our results thus remain the only source of evidence.

The increased postoperative mortality in patients with liver cirrhosis may have several explanations. Liver cirrhosis is a complex disease involving different organ systems, increasing the risk of postoperative complications, and decreasing the patient’s recuperative capacity [4]. Previous studies have identified hepatic coagulopathy as a risk factor for postoperative mortality in patients with chronic liver disease undergoing surgery [22-24], as well as ascites, hepatic encephalopathy, elevated creatinine levels, and other manifestations of portal hypertension [25]. Furthermore, liver disease is known to modify the effect of various drugs, attenuate immune function, and consequently increase the risk of infection and eventually mortality [4,26,27]. Finally, although liver disease, especially liver cirrhosis, has negative systemic effects, other diseases coexisting with liver disease may also contribute to increased postoperative mortality as suggested by the less pronounced impact of liver disease in patients with severe comorbidity.

The validity of our findings depends on several factors. We used population-based registries with complete follow-up. We had complete data on surgical procedures and on hospital diagnoses, which minimized selection and referral bias. Both the DCR data on cancer [12] and the DNRP [13] data on liver diseases, surgical procedures and comorbidity [17] are of high quality. Nonetheless, we cannot rule out that our results were affected by undiagnosed liver diseases, but this would have caused us to underestimate the RRs of postoperative mortality. Moreover, we included patients with both acute and chronic non-cirrhotic liver disease, such as viral hepatitis, in the non-cirrhotic liver disease cohort. Hence, it is likely that some patients had completely recovered from an acute liver disease by the time of surgery. We may therefore have underestimated the impact of non-cirrhotic liver disease on mortality.

## Conclusion

Our data show that patients with liver disease, especially liver cirrhosis, have markedly increased mortality after CRC surgery compared to patients without liver disease. Perioperative management of patients with liver disease should thus be carefully planned in order to minimize complications and death.

## Abbreviations

CI: Confident Interval; CPR number: Unique Civil Registration Number; CRC: Colorectal Cancer; DCR: Danish Cancer Registry; DNRP: Danish National Registry of Patients; ICD: International Classification of Diseases; NOMESCO: Nordic Medico-Statistical Committee; RR: Relative Risk.

## Competing interests

The authors disclose no competing interests.

## Authors’ contributions

JM: study concept and design, data interpretation, and manuscript preparation; RE, CFC: study concept and design, data interpretation, and manuscript review; SPU, LP: acquisition of data, and statistical analysis; TN, HTS: study design, critical analysis of the data, manuscript review, and study supervision. All the authors have approved the final draft submitted.

## Pre-publication history

The pre-publication history for this paper can be accessed here:

http://www.biomedcentral.com/1471-230X/13/66/prepub

## Supplementary Material

Additional file 1 Codes used in the analysis. Click here for file

Additional file 2 Descriptive table on distribution of comorbidity depending on cancer stage in patients with no liver disease, non-cirrhotic liver disease, and liver cirrhosis. Click here for file

Additional file 3 Relative risk (RR) and 30-day mortality after acute and elective colorectal cancer surgery in patients without liver disease, in those with non-cirrhotic liver disease, and in those with liver cirrhosis. Click here for file

## References

[B1] Sorensen HT Friis S Olsen JH Thulstrup AM Mellemkjaer L Linet M Trichopoulos D Vilstrup H Olsen J Risk of liver and other types of cancer in patients with cirrhosis: a nationwide cohort study in Denmark Hepatology 1998 28 4 921 925 10.1002/hep.510280404 9755226

[B2] Sorensen HT Mellemkjaer L Jepsen P Thulstrup AM Baron J Olsen JH Vilstrup H Risk of cancer in patients hospitalized with fatty liver: a Danish cohort study J Clin Gastroenterol 2003 36 4 356 359 10.1097/00004836-200304000-00015 12642745

[B3] Volet J de Mestier L Ehrhard F Bouche O News in management of colorectal cancer at JFHOD 2012 meeting Bull Cancer 2012 99 6 703 713 22652258 10.1684/bdc.2012.1588

[B4] Wiklund RA Preoperative preparation of patients with advanced liver disease Crit Care Med 2004 32 4 Suppl S106 S115 15064669 10.1097/01.ccm.0000115624.13479.e6

[B5] Gervaz P Pak-art R Nivatvongs S Wolff BG Larson D Ringel S Colorectal adenocarcinoma in cirrhotic patients J Am Coll Surg 2003 196 6 874 879 10.1016/S1072-7515(03)00117-0 12788423

[B6] Ghaferi AA Mathur AK Sonnenday CJ Dimick JB Adverse outcomes in patients with chronic liver disease undergoing colorectal surgery Ann Surg 2010 252 2 345 350 10.1097/SLA.0b013e3181e982d6 20622652

[B7] Csikesz NG Nguyen LN Tseng JF Shah SA Nationwide volume and mortality after elective surgery in cirrhotic patients J Am Coll Surg 2009 208 1 96 103 10.1016/j.jamcollsurg.2008.09.006 19228510

[B8] Metcalf AM Dozois RR Wolff BG Beart RW Jr The surgical risk of colectomy in patients with cirrhosis Dis Colon Rectum 1987 30 7 529 531 10.1007/BF02554783 3595374

[B9] Meunier K Mucci S Quentin V Azoulay R Arnaud JP Hamy A Colorectal surgery in cirrhotic patients: assessment of operative morbidity and mortality Dis Colon Rectum 2008 51 8 1225 1231 10.1007/s10350-008-9336-y 18521677

[B10] Nguyen GC Correia AJ Thuluvath PJ The impact of cirrhosis and portal hypertension on mortality following colorectal surgery: a nationwide, population-based study Dis Colon Rectum 2009 52 8 1367 1374 10.1007/DCR.0b013e3181a80dca 19617746

[B11] Frank L Epidemiology. When an entire country is a cohort Science 2000 287 5462 2398 2399 10.1126/science.287.5462.2398 10766613

[B12] Gjerstorff ML The danish cancer registry Scand J Public Health 2011 39 Suppl 7 42 45 21775350 10.1177/1403494810393562

[B13] Lynge E Sandegaard JL Rebolj M The Danish National Patient Register Scand J Public Health 2011 39 Suppl 7 30 33 21775347 10.1177/1403494811401482

[B14] Health Classifications in the Nordic Countries http://nomesco-eng.nom-nos.dk/filer/publikationer/KlassifikationshistorieWeb.pdf

[B15] Sogaard KK Horvath-Puho E Gronbaek H Jepsen P Vilstrup H Sorensen HT Risk of venous thromboembolism in patients with liver disease: a nationwide population-based case-control study Am J Gastroenterol 2009 104 1 96 101 10.1038/ajg.2008.34 19098856

[B16] Charlson ME Pompei P Ales KL MacKenzie CR A new method of classifying prognostic comorbidity in longitudinal studies: development and validation J Chronic Dis 1987 40 5 373 383 10.1016/0021-9681(87)90171-8 3558716

[B17] Thygesen SK Christiansen CF Christensen S Lash TL Sorensen HT The predictive value of ICD-10 diagnostic coding used to assess Charlson comorbidity index conditions in the population-based Danish National Registry of Patients BMC Med Res Methodol 2011 11 83 10.1186/1471-2288-11-83 21619668 PMC3125388

[B18] Sorensen LT Jorgensen T Kirkeby LT Skovdal J Vennits B Wille-Jorgensen P Smoking and alcohol abuse are major risk factors for anastomotic leakage in colorectal surgery Br J Surg 1999 86 7 927 931 10.1046/j.1365-2168.1999.01165.x 10417567

[B19] Pedersen CB Gotzsche H Moller JO Mortensen PB The Danish Civil Registration System. A cohort of eight million persons Dan Med Bull 2006 53 4 441 449 17150149

[B20] Sorensen HT Thulstrup AM Mellemkjar L Jepsen P Christensen E Olsen JH Vilstrup H Long-term survival and cause-specific mortality in patients with cirrhosis of the liver: a nationwide cohort study in Denmark J Clin Epidemiol 2003 56 1 88 93 10.1016/S0895-4356(02)00531-0 12589875

[B21] Christensen S Johansen MB Pedersen L Jensen R Larsen KM Larsson A Tonnesen E Christiansen CF Sorensen HT Three-year mortality among alcoholic patients after intensive care: a population-based cohort study Crit Care 2012 16 1 R5 10.1186/cc10603 22226344 PMC3396230

[B22] Ziser A Plevak DJ Wiesner RH Rakela J Offord KP Brown DL Morbidity and mortality in cirrhotic patients undergoing anesthesia and surgery Anesthesiology 1999 90 1 42 53 10.1097/00000542-199901000-00008 9915311

[B23] Mansour A Watson W Shayani V Pickleman J Abdominal operations in patients with cirrhosis: still a major surgical challenge Surgery 1997 122 4 730 735 10.1016/S0039-6060(97)90080-5 9347849

[B24] Rice HE O'Keefe GE Helton WS Johansen K Morbid prognostic features in patients with chronic liver failure undergoing nonhepatic surgery Arch Surg 1997 132 8 880 884 10.1001/archsurg.1997.01430320082013 9267273

[B25] Rizvon MK Chou CL Surgery in the patient with liver disease Med Clin North Am 2003 87 1 211 227 10.1016/S0025-7125(02)00153-0 12575891

[B26] Malik SM Ahmad J Preoperative risk assessment for patients with liver disease Med Clin North Am 2009 93 4 917 929 10.1016/j.mcna.2009.03.001 19577122

[B27] Patel T Surgery in the patient with liver disease Mayo Clin Proc 1999 74 6 593 599 10.4065/74.6.593 10377935

