# Supplementary material for: Liver disease and 30-day mortality after colorectal cancer surgery: a Danish population-based cohort study
Source: BMC Gastroenterol. 2013 Apr 15;13:66. doi: 10.1186/1471-230X-13-66 (PMC3637330; doi:10.1186/1471-230X-13-66)
Supplement: Additional file 1 — Codes used in the analysis. [file 1471-230X-13-66-S1.doc]

**Additional file 1 – Codes used in the analysis**

ICD codes, NOMESCO codes, and cancer stage codes used in the analysis, are shown.

***The Danish Cancer Registry***

*Colorectal Cancer:* ICD-8: 153-154; ICD-10: C18, C19, C20

*Colorectal cancer stage classification:*

- Localized:
  - Dukes: A,B
  - TNM*: T0,1-4,x N0 M0; T0,1-2 N0 Mx; T0,1 Nx M0,x
- Non-localized:
  - Dukes: C,D
  - TNM*: T0,1-4,x N1-3 M0-1,x; T0,1-4,x N0 M1; T0,1-4,x Nx M1
- Unknown
  - TNM*: T2-4,x Nx M0,x; T2-4,x N0 Mx

* Colorectal cancers were classified according to TNM from 2004 on.

***The Danish National Registry of Patients***

Hospital diagnoses were as follows:

- *Colorectal surgery (NOMESCO codes):*
  - Open radical resection: JGB00, JGB10,JGB20, JGB30, JGB40, JGB50, JGB60, JGB96, JFB20, JFB30, JFB 33, JFB40, JFB43, JFB46, JFB50, JFB60, JFB63, JFB96, JFH00, JFH10, JFH20, JFH30, JFH33, JFH40, JFH96, JGA00, JGA70
  - Laparoscopic radical resection: JGB01, JGB11, JGB31, JGB97, JFB21, JFB31, JFB34, JFB41, JFB44, JFB47, JFB51, JFB61, JFB64, JFB97, JFH01, JFH11
  - Non-eradicative procedures: JGA32-58, JGA73-98, JGW, JFA68, JFA83-84, JFA96-97, JFC, JFF10-13, JFF20-31, JFW
- *Liver cirrhosis*: ICD-8: 571.09, 571.92, 571.99; ICD-10: K70.3, K71.7, K74.5, K74.6
- *Non-cirrhotic liver disease* 🡪 ICD-8: 570.00–573.09 (excluding 571.09, 571.92, 571.99), 070.01-070.09; ICD-10: K70.0- K70.9 (excluding K70.3) R74.0, K71.0–K77.8 (excluding K71.7, K74.5, K74.6), B15–B19
- *Disease included in the adjusted Charlson Comorbidity Index:*
  - Myocardial infarction: ICD-8: 410; ICD-10: I21, I22, I23
  - Congestive heart failure: ICD-8: 427.09, 427.10, 427.11, 427.19, 428.99, 782.49; ICD-10: I50, I11.0, I13.0, I13.2
  - Peripheral vascular disease: ICD-8: 440, 441, 442, 443, 444, 445; ICD-10: I70, I71, I72, I73, I74, I77
  - Cerebrovascular disease: ICD-8: 430-438; ICD-10: I60-I69, G45, G46
  - Dementia: ICD-8: 290.09-290.19, 293.09; ICD-10: F00-F03, F05.1, G30
  - Chronic pulmonary disease: ICD-8: 490-493, 515-518; ICD-10: J40-J47, J60-J67, J68.4, J70.1, J70.3, J84.1, J92.0, J96.1, J98.2, J98.3
  - Connective tissue disease: ICD-8: 712, 716, 734, 446, 135.99; ICD-10: M05, M06, M08, M09, M30, M31, M32, M33, M34, M35, M36, D86
  - Ulcer disease: ICD-8: 530.91, 530.98, 531-534; ICD-10: K22.1, K25-K28
  - Uncomplicated type 1 and type 2 diabetes: ICD-8: 249.00, 249.06, 249.07, 249.09, 250.00, 250.06, 250.07, 250.09; ICD-10: E10.0, E10.1, E10.9, E11.0, E11.1, E11.9
  - Hemiplegia: ICD-8: 344; ICD-10: G81, G82
  - Moderate to severe renal disease: ICD-8: 403, 404, 580-583, 584, 590.09, 593.19, 753.10-753.19, 792; ICD-10: I12, I13, N00-N05, N07, N11, N14, N17-N19, Q61
  - Diabetes with end-organ damage: ICD-8: 249.01-249.05, 249.08, 250.01-250.05, 250.08; ICD-10: E10.2-E10.8, E11.2-E11.8
  - Any tumor (excluding CRC): ICD-8: 140-194 (excluding 153-154, 155.09); ICD-10: C00-C75 (excluding C18-C20, C22)
  - Leukemia: ICD-8: 204-207; ICD-10: C91-C95
  - Lymphoma: ICD-8: 200-203, 275.59; ICD-10: C81-C85, C88, C90, C96
  - Metastatic solid tumor (excluding metastases from CRC): ICD-8: 195-198, 199 (excluding patients with diagnoses 197.59, 197.79, and 197.89); ICD-10: C76-C80 (excluding patients with diagnoses C78.5 and C78.7)
  - AIDS: ICD-8: 079.83; ICD-10: B21-B24
- *Non-hepatic alcohol-related disease:* ICD-8: 291-291.9, 303-303.9, 980; ICD-10: F10.2, F10.7, F10.8, I42.6, G62.1, K29.2, G72.1, G31.2, T51, Z72.1
- *Gastric and esophageal varices:* ICD-8: 456.0X; ICD-10: I85.X, I86.4
