# Supplementary material for: Liver disease and 30-day mortality after colorectal cancer surgery: a Danish population-based cohort study
Source: BMC Gastroenterol. 2013 Apr 15;13:66. doi: 10.1186/1471-230X-13-66 (PMC3637330; doi:10.1186/1471-230X-13-66)
Supplement: Additional file 2 — Descriptive table on distribution of comorbidity depending on cancer stage in patients with no liver disease, non-cirrhotic liver disease, and liver cirrhosis. [file 1471-230X-13-66-S2.docx]

**Additional file 2**

Descriptive table on distribution of comorbidity depending on cancer stage in patients with no liver disease, non-cirrhotic liver disease, and liver cirrhosis.

| **Cancer stage** | **No liver disease**  **N (%)**  **n = 39,313** | **Non-cirrhotic liver disease**  **N (%)**  **n = 369** | **Liver cirrhosis**  **N (%)**  **n = 158** |
| --- | --- | --- | --- |
| **Localized:**   - Low comorbidity level - Moderate comorbidity level - High comorbidity level | 17,044 (43.4%)  10,486 (26.7%)  5,109 (13.0%)  1,449 (3.7%) | 163 (44.2%)  75 (20.3%)  67 (18.2%)  21 (5.7%) | 65 (41.1%)  29 (18.4%)  26 (16.4%)  10 (6.3%) |
| **Non-localized:**   - Low comorbidity level - Moderate comorbidity level - High comorbidity level | 18,863 (48.0%)  11,993 (30.5%)  5,298 (13.5%)  1,572 (4.0%) | 182 (49.3%)  87 (23.5%)  63 (17.1%)  32 (8.7%) | 76 (48.1)  26 (16.5%)  31 (19.6%)  19 (12.0%) |
| **Stage unknown:**   - Low comorbidity level - Moderate comorbidity level - High comorbidity level | 3,406 (8.6%)  1,822 (4.6%)  1,166 (3.0%)  418 (1.0%) | 24 (6.5%)  5 (1.4%)  15 (4.0%)  4 (1.1%) | 17 (10.8%)  5 (3.2%)  8 (5.1%)  4 (2.5%) |
