# Supplementary material for: Liver disease and 30-day mortality after colorectal cancer surgery: a Danish population-based cohort study
Source: BMC Gastroenterol. 2013 Apr 15;13:66. doi: 10.1186/1471-230X-13-66 (PMC3637330; doi:10.1186/1471-230X-13-66)
Supplement: Additional file 3 — Relative risk (RR) and 30-day mortality after acute and elective colorectal cancer surgery in patients without liver disease, in those with non-cirrhotic liver disease, and in those with liver cirrhosis. [file 1471-230X-13-66-S3.doc]

**Additional file 3**

Relative risk (RR) and 30-day mortality after acute and elective colorectal cancer surgery in patients without liver disease, in those with non-cirrhotic liver disease, and in those with liver cirrhosis.

| **Cancer site** | **Patients**  **N** | **Deaths within 30 days**  **N** | **30-day mortality %***  **(95% CI)** | **RR (95% CI)** | |
| --- | --- | --- | --- | --- | --- |
|  |  |  |  | **Crude** | **Adjusted** |
| **Acute surgery^¥^**   - No liver disease - Non-cirrhotic liver disease   - Liver cirrhosis | 12,633  137  59 | 2064  33  21 | 16.3 (15.6-17.1)  24.1 (16.3-34.7)  35.6 (20.9-56.2) | 1.00  1.56 (1.10-2.20)  2.38 (1.55-2.67) | 1.00  1.57 (1.11-2.22)  2.48 (1.59-3.88) |
| **Elective surgery^¥^**   - No liver disease - Non-cirrhotic liver disease   - Liver cirrhosis | 26,602  231  99 | 1363  16  17 | 5.1 (4.9-5.4)  6.9 (4.2-11.4)  17.2 (10.2-28.1) | 1.00  1.36 (0.83-2.23)  3.49 (2.16-5.63) | 1.00  1.39 (0.85-2.28)  2.79 (1.70-4.57) |

* Calculated using the Kaplan-Meier method.

**^¥^** Information on surgery timing is missing for some patients therefore the sum of patients undergoing to acute and elective surgery is not equal to the number of all patients included in the study.
